# Human junctophilin-2 undergoes a structural rearrangement upon binding PtdIns(3,4,5)*P*_3_ and the S101R mutation identified in hypertrophic cardiomyopathy obviates this response

**DOI:** 10.1042/BJ20130591

**Published:** 2013-11-08

**Authors:** Hayley J. Bennett, John Bernard Davenport, Richard F. Collins, Andrew W. Trafford, Christian Pinali, Ashraf Kitmitto

**Affiliations:** *Institute of Cardiovascular Sciences, Faculty of Medical and Human Sciences, University of Manchester, Manchester M13 9NT, U.K.; †Michael Smith Building, Faculty of Life Sciences, University of Manchester, Manchester M13 9LT, U.K.

**Keywords:** junctophilin-2, phosphatidylserine, PtdIns(3,4,5)*P*_3_, quartz-crystal microbalance with dissipation monitoring (QCM-D), supported lipid bilayer, CICR, calcium-induced calcium release, DOPC, 1,2-dioleoyl-*sn*-glycero-3-phosphocholine, DOPE, 1,2-dioleoyl-*sn*-glycero-3-phosphoethanolamine, DOPG, 1,2-dioleoyl-*sn*-glycero-3-phosphoglycerol, DOPS, 1,2-dioleoyl-*sn-*glycero-3-phospho-L-serine, EM, electron microscopy, HCM, hypertrophic cardiomyopathy, JP, junctophilin, jSR, junctional sarcoplasmic reticulum, MBP, maltose-binding protein, MORN, membrane occupation and recognition nexus, PA, phosphatidic acid, PC, phosphatidylcholine, PH, pleckstrin homology, PI3K, phosphoinositide 3-kinase, POPC, 1-palmitoyl-2-oleoyl-*sn*-glycero-3-phosphocholine, PS, phosphatidylserine, QCM-D, quartz-crystal microbalance with dissipation monitoring, RyR, ryanodine receptor, SLB, supported lipid bilayer, SM, sphingomyelin, SUV, small unimellar vesicle, TEM, transmission electron microscopy, t-t, transverse-tubule

## Abstract

JP2 (junctophilin-2) is believed to hold the transverse tubular and jSR (junctional sarcoplasmic reticulum) membranes in a precise geometry that facilitates excitation–contraction coupling in cardiomyocytes. We have expressed and purified human JP2 and shown using electron microscopy that the protein forms elongated structures ~15 nm long and 2 nm wide. Employing lipid-binding assays and quartz crystal microbalance with dissipation we have determined that JP2 is selective for PS (phosphatidylserine), with a *K*_d_ value of ~0.5 μM, with the N-terminal domain mediating this interaction. JP2 also binds PtdIns(3,4,5)*P*_3_ at a different site than PS, resulting in the protein adopting a more flexible conformation; this interaction is modulated by both Ca^2+^ and Mg^2+^ ions. We show that the S101R mutation identified in patients with hypertrophic cardiomyopathy leads to modification of the protein secondary structure, forming a more flexible molecule with an increased affinity for PS, but does not undergo a structural transition in response to binding PtdIns(3,4,5)*P*_3_. In conclusion, the present study provides new insights into the structural and lipid-binding properties of JP2 and how the S101R mutation may have an effect upon the stability of the dyad organization with the potential to alter JP2–protein interactions regulating Ca^2+^ cycling.

## Abstract 2

We have purified human JP2 (junctophilin-2) and the S101R hypertrophic cardiomyopathy mutant. JP2 interacts with phosphatidylserine, *K*_d_ ~0.5 μM, and PtdIns(3,4,5)*P*_3_ at different sites; divalent cations perturb the association. S101R has a modified structure and phospholipid-binding properties.

## INTRODUCTION

Cardiac contraction is regulated by Ca^2+^ through a process termed CICR (calcium-induced calcium release). Within the myocyte t-ts (transverse-tubules) and the jSR (junctional sarcoplasmic reticulum) membranes form a microdomain termed a dyad [1]. The two membranes are separated by a gap of ~12–15 nm [2–4]. Disruption of the dyad architecture results in partial uncoupling of the geometric relationship between two of the protein components governing CICR: the sarcolemmal LTCCs (L-type voltage-gated calcium channels) and RyRs (ryanodine receptors) in the jSR, resulting in perturbed Ca^2+^ dynamics, which is a hallmark of heart failure [5]. Both a gradual drift of the t-t membranes [6] and an expansion of the dyadic cleft [7] have been proposed to lead to the loss of the dyad Ca^2+^ microdomain and the spatial and temporal restrictions that maintain CICR. However, the molecular mechanisms that underlie this cellular re-organization remain poorly understood.

JP2 (junctophilin-2), a member of the JP family [8], has been identified in cardiac myocytes as a ‘linker’ protein spanning and holding the two membrane systems in a precise geometry to facilitate CICR. It is thought that the C-terminus of JP2 is anchored within the sarcoplasmic reticulum membrane with the molecule extending across the dyadic space so that the N-terminal portion is able to bind to the t-t membrane via a unique structural MORN (membrane occupation and recognition nexus) domain. The MORN motif (YQ/EGE/QT-X-NGK-X-HGYG) was first identified in JP1 in murine skeletal muscle [9] and it was suggested that these domains adhere to the t-t through an interaction with SM (sphingomyelin) and PC (phosphatidylcholine); to date there is no direct experimental data to describe the molecular basis for the association of JP2 with membrane lipids. Moreover, there is no structural data for any of the JPs.

A relationship between the down-regulation of JP2 and cardiac pathologies is now supported by a number of studies. For example, Wei et al. [10] have shown in a mouse model of heart failure, induced by thoracic aortic banding, that there is a loss of JP2 expression and t-t membrane remodelling. Cardiac-specific JP2-knockout mice are reported to develop heart failure with a depleted number of intact dyad structures [11]. Studies of a mouse model of cardiomyopathy identified JP2 down-regulation, by up to 60% (mRNA level), accompanied by depressed [Ca^2+^]_i_ transients [12]. Similarly, a reduction in JP2 expression (~50%) has been described in rats with pressure overload-induced hypertrophy with a desynchronized Ca^2+^ spark profile [13]. Hence there is a clear link emerging between JP2 expression levels, dyad disruption and a loss of t-t integrity resulting in disturbed calcium homoeostasis.

Four mutations of the JP2 gene (*JPH2*), S101R, Y141H, S165F and G505S, have also been identified in patients with HCM (hypertrophic cardiomyopathy) [14,15]. Significantly, no other mutations of currently identified HCM-linked genes were identified in these patients, thereby suggesting that JP2 is a key mediator in the pathogenesis of disease. It has been shown that Y141H and S165F expressed in the cardiomyoblast cell line HC92 leads to a 2–3-fold increase in cell size [16], whereas transfection of the S101R mutant did not lead to any significant change to the cell dimensions. The molecular basis for why the S101R mutation leads to disease is yet to be elucidated.

The aim of the present study was to express and purify JP2 in order to investigate the mechanisms by which the protein associates with lipids commonly found in mammalian cells [17]. We extended our studies to also examine whether the introduction of the S101R mutation identified in HCM patients alters the lipid-binding properties of the protein, a feature that may affect the stability of the dyadic cleft.

## EXPERIMENTAL

### Expression and purification of JP2

The ORF of *JPH2*, encoding full-length JP2 (NCBI accession number NP-065166.2), and a truncated form (residues 1–452) were PCR amplified from human heart cDNA and cloned into a modified pMalC2X plasmid (GE Healthcare) to generate an N-terminal PreScission™ protease cleavable MBP (maltose-binding protein)-tagged expression construct. Site-directed mutagenesis of S101R was carried out using the QuikChange® site-directed mutagenesis kit (Stratagene). All constructs were verified by DNA sequencing (GATC Biotech). Each construct was transformed into *Escherichia coli* BL21 cells and at an *D*_600_ of ~0.6 expression was induced using 0.25 mM IPTG, followed by incubation at 16°C for 16 h. The recombinant MBP-tagged proteins were purified by amylose affinity chromatography. The MBP tag was cleaved from JP2 by incubation with PreScission protease (GE Healthcare) for 18 h at 4°C, followed by size-exclusion chromatography (ÄKTA FPLC) using a Superdex 200 10/300GL column in 25 mM Tris, 150 mM NaCl and 1 mM DTT (pH 7.4). Purification was confirmed by SDS/PAGE and verified using Western blotting (Abcam ab116077, detects JP2 N-terminal domain and Abcam ab79071, detects C-terminal domain). Protein yields were in the range of 1 mg/l of *E. coli* in a final buffer of 25 mM Tris/HCl, pH 7.4, and 150 mM NaCl.

### Protein–lipid overlay assays

Lipid aliquots (400 pmol) were adsorbed and dried on to nitrocellulose paper and protein was then applied (2 μg/ml) with incubation for 1 h. The lipid strips were then developed following standard protocols for FAT Western blotting using the primary antibodies ab79071 and ab116077 with alkaline phosphatase conjugated to the secondary antibody. PIP strips™ and PIP arrays (Invitrogen) were employed using the manufacturer's instructions.

### Formation of SUVs (small unimellar vesicles)

The lipids DOPC (1,2-dioleoyl-*sn*-glycero-3-phosphocholine), DOPE (1,2-dioleoyl-*sn*-glycero-3-phosphoethanolamine), DOPS (1,2-dioleoyl-*sn-*glycero-3-phospho-L-serine) and POPC (1palmitoyl-2-oleoyl-*sn*-glycero-3-phosphocholine) were purchased from Sigma, resuspended in chloroform to create 2 mM stocks and PtdIns(3,4,5)*P*_3_ was resuspended in chloroform/methanol/water (5:10:4, by vol.) at 0.22 mM. The lipids were mixed for molar ratios of DOPC/DOPE/POPC/DOPS (55:15:10:20, by vol.), DOPC/DOPE/POPC/DOPS/PtdIns(4,5)*P*_2_ (55:15:9:20:1, by vol.) and DOPC/DOPE/POPC/DOPS/PtdIns(3,4,5)*P*_3_ (55:15:9:20:1, by vol.), dried under nitrogen for 30 mins, placed under vacuum for 1 h at room temperature (23°C) and then stored under nitrogen at −20°C. The lipids were hydrated in 20 mM Tris/HCl, pH 7.4, and 150 mM NaCl and vortex-mixed (6×15 s) followed by alternating 10 min of incubation at 37°C and at room temperature. SUVs were then formed by 21 passes of the hydrated lipids through a mini-extruder (Avanti Polar Lipids) fitted with a 0.05 μm filter (Whatman). The SUVs were stored under nitrogen at 4°C prior to use. Light scattering showed that the vesicles were typically between 80–100 nm in diameter.

### The QCM-D technique, the formation of SLBs (supported lipid bilayers) and protein–lipid binding studies

QCM-D (quartz-crystal microbalance with dissipation monitoring) is an acoustic technique using sensor (quartz crystal) technology developed on the basis of Sauerbrey's theory [18]. The application of an AC voltage leads to oscillation of the quartz crystal at its fundamental frequency *f*. QCM-D can be considered to be a nano-sensitive weighing device whereby the addition of mass to the surface of the chip leads to a change in the frequency (*f*) which is directly proportional to the mass [19]. The dissipation measurement, *D*, is correlated to the viscous loss or flexibility of the sample, for example a small Δ*D* is generally associated with a compact rigid layer. Multiple harmonics are monitored for both *f* and *D* with the greater the spread of the dissipation overtones, the more soft or loose the sample is on the chip.

SLBs were formed on a silicon oxide (SiO_2_) chip using the Q-sense E1 system (Biolin Scientific), with a fundamental resonance frequency of 5 MHz. The SiO_2_ chip was cleaned by sonication in 2% SDS, rinsed in water and ethanol, dried in a nitrogen stream, and given 20 min UV-ozone treatment (UV/Ozone ProCleaner, BioForce Nanosciences). The chip was placed in the QCM-D chamber and the signal was stabilized in 20 mM Tris/HCl, pH 7.4, and 150 mM NaCl across the chip with a flow rate of 500 μl/min for 30–60 min. The SUVs (composition as above) were diluted in the same buffer for a final concentration of 150 μg/ml; 500 μl of this dilution was applied at a flow-rate of 50 μl/min and 28°C to form the SLB [20]. The integrity of the SLB was tested by applying 50 μg/ml RNase A in the same buffer, the absence of a change in frequency was taken to indicate that the SLB had formed across the entire chip, since RNase A does not bind to lipids, but would be able to occupy any spaces not covered by the bilayer on the sensor chip. A salt wash was then applied and the SLB was equilibrated in the JP2 sample buffer (20 mM Tris/HCl, pH 7.4, and 150 mM NaCl at a flow rate of 50 μl/min for 30–60 min prior to application of the protein).

Aliquots of JP2 (100 μl of a 200 nM JP2 solution) at a flow rate of 50 μl/min were sequentially added, maintaining the same flow rate until there was no further change to the frequency, which was taken to indicate that the there was full coverage of the SLB, i.e. all binding sites were occupied. For each addition of protein, the frequency change Δ*f* (*f*_max_−*f*_0_) was plotted against JP2 concentration. To compare results between experiments, we normalized the data and plotted [(*f*_max_−*f*_0_)/*f*_max_]. The change in frequency was measured at the third overtone where *f*_0_ is taken as the stable frequency of the formed SLB (typically between −25 to −30 Hz). A frequency shift of ~1 Hz corresponds to a mass change of ~6 ng/cm^2^ (Saubrey's equation). All experiments were conducted in triplicate. We employed GraphPad Prism to model the isotherms as described in [21] to determine the dissociation constant *K*_d_:

$$K_{d}^{-1}•C=\Theta /\Phi \left(\Theta \right)$$

where *C* is the protein concentration and Θ is the surface coverage which fits a one-site binding model. The function Φ(Θ) is the available surface. It is assumed that binding to individual phospholipids is non-co-operative and that the adsorption energy at each binding site is the same for the protein (adsorbate). The Hill coefficient (*h*) describes the co-operativity of binding where values of 1 indicate non-co-operativity, and when *h*>1 there is positive co-operativity and *h*<1 there is negative co-operativity. The magnitude of the dissipation change (Δ*D*) upon addition of the protein to the SLB was taken to be the difference between overtone D_13_ for the SLB subtracted from D_13_ for the protein once saturation had been reached. The spread of the dissipation overtones is the difference between overtone *D*_3_ and *D*_13_. *D*−*f* graphs were calculated by plotting the change in frequency and dissipation with values taken from the point where the protein was added to the bilayer. Data are reported as means±S.D. and data comparisons were made using one-way ANOVA or Student's *t* test as appropriate.

### JP2 secondary structure analysis

Protein samples (2 μM) were examined in a Jasco J810 CD spectrometer with a peltier temperature controller set to 20°C. Wavelength scans were performed using a 0.5 mm-path-length and data were collected every 0.2 nm with a 1 nm bandwidth. Each spectrum was obtained from the accumulation of data from 12 scans; baseline was corrected using the spectrum of a Tris/NaCl buffer blank. Data were collected between 190 nm and 260 nm. The CD signal for JP2 and S101R were deconvoluted using DichroWeb [22,23] applying the CDSSTR algorithm.

### Electron microscopy

Purified JP2 was diluted to concentrations employed for the Q-sense experiments (15 μg/ml) negatively stained using standard protocols with 2% (w/v) uranyl acetate and examined using Tecnai 12 TEM (transmission electron microscopy) operated at 100 kV. Micrographs of JP2 were recorded using a 4K×4K CCD (charge-coupled-device) Gatan camera with a calibrated magnification corresponding to 3.5 Å (1 Å=0.1 nm)/pixel at the specimen level. Particles were selected using the software boxer part of the EMAN suite [24].

## RESULTS

**Figure 1:**
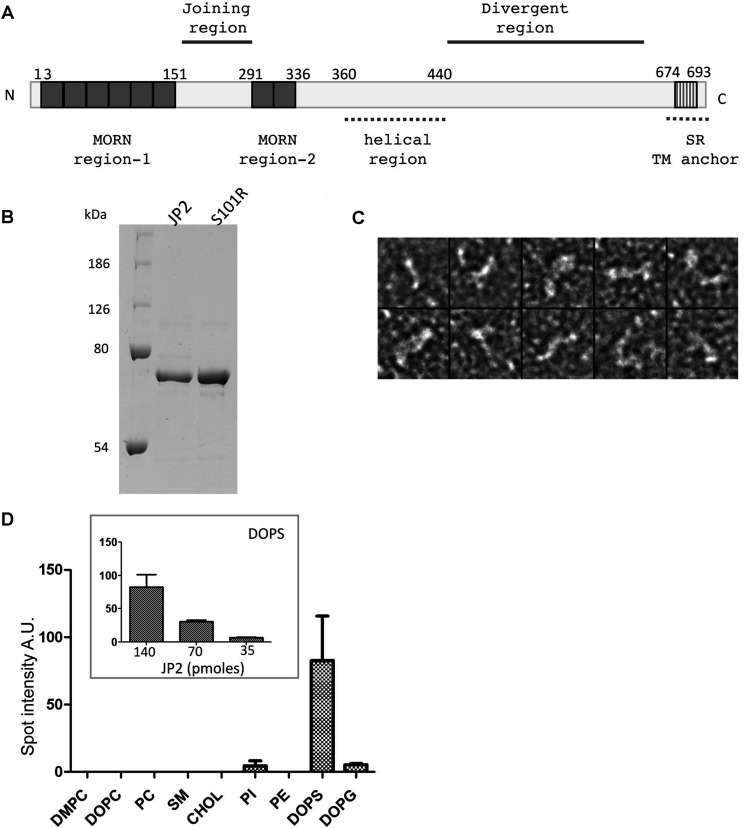
Purification of JP2 and analysis of lipid-binding characteristics (**A**) Putative domains of JP2 based on the primary sequence. (**B**) Coomassie-Blue-stained SDS/PAGE (10% gel) of purified wild-type JP2 and mutant S101R showing a single polypeptide band at ~75 kDa. Molecular masses are indicated in kDa. (**C**) TEM images of negatively stained JP2 showing that the protein forms filament structures ~15 nm in length and 2 nm in diameter; box size=25 × 25 nm. (**D**) Densitometric analysis of lipid–protein overlay (*n*=3). The inset illustrates that the binding of JP2 to DOPS is quantitative. A.U., absorbance units; CHOL, cholesterol; PI, PtdIns; SR, sarcoplasmic reticulum; TM, transmembrane.

In the present study we describe the first overexpression of soluble tagged full-length human JP2 in *E. coli* which we have purified to homogeneity using column chromatography, with the SDS/PAGE gel in Figure 1(B) showing a single polypeptide band at ~75 kDa. Examination of purified JP2 by TEM after negative staining revealed that the protein forms an elongated structure that is approximately 15 nm in length and 2 nm thick. The montage in Figure 1(C) shows that, upon absorption to the carbon film, a variety of orientations of a filament-type structure are captured, some are rod shaped, whereas others have bends and kinks.

### Binding of recombinant JP2 to anionic phospholipids

**Figure 2:**
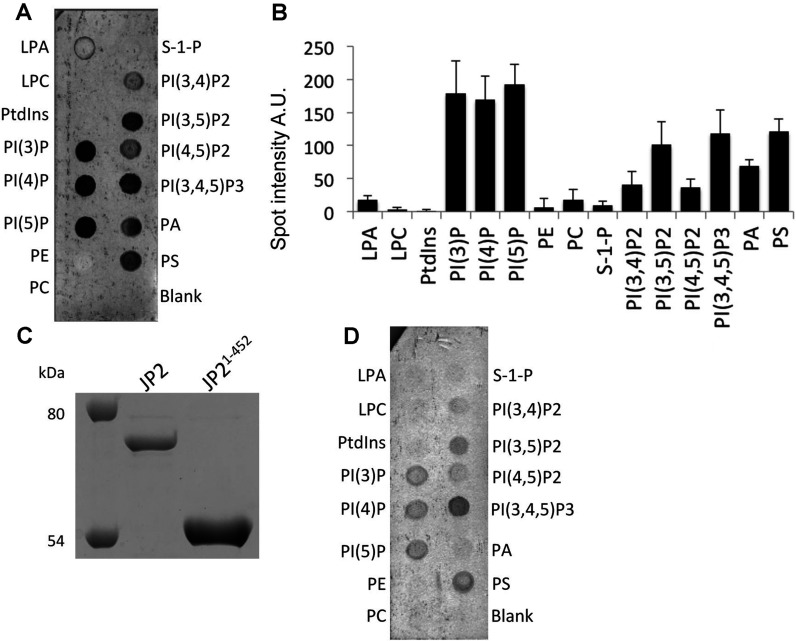
Full-length JP2 and the N-terminal domain (JP2^1–452^) bind to phosphatidylinositols (**A**) Examplar PIP strip™ revealing that JP2 also binds to phosphatidylinositols (primary antibody ab79071). (**B**) Densitometric analysis of JP2 binding to PIP strips™ (*n*=3). (**C**) Coomassie-Blue-stained SDS/PAGE (10% gel) illustrating that JP2^1–452^ was isolated as a single polypeptide band at ~50 kDa. The full-length protein is also shown for comparative purposes. Molecular masses are indicated in kDa. (**D**) JP2^1–452^ binding to PIP strip™ (primary antibody ab116077). A.U., absorbance units; LPA, lysophosphatidic acid; LPC, lysophosphocholine; PI, PtdIns; S-1-P, sphingosine 1-phosphate.

Studies of the the lipid-binding properties of JP2, using protein–lipid overlay assays, revealed that JP2 binds most strongly to DOPS (Figure 1D), with no detectable interaction with SM or PC. Studies of a MORN domain within the plant *Arabidopsis* phosphatidylinositol phosphate kinase 1 found that this region of the enzyme bound to phosphatidylinositols, specifically PtdIns4*P* and PtdIns(4,5)*P*_2_ [25]. We therefore extended our studies to examine the interaction of JP2 with an array of mono-, di- and tri-phosphoinositols using PIP strips™. JP2 binds to the monophosphates PtdIns3*P*, PtdIns4*P* and PtdIns5*P*, the biphosphate PtdIns(3,5)*P*_2_, the triphosphate PtdIns(3,4,5)*P*_3_, and to a lesser extent to PtdIns(4,5)*P*_2_ (Figures 2A and 2B). Since there is no interaction of the protein with PtdIns, we can conclude that the addition of the phosphate group to the lipid is important for mediating an interaction with JP2. The protein also binds to PA (phosphatidic acid) and PS (phosphatidylserine).

Since the hydrophobic C-terminal domain of the full-length JP2 may be responsible for mediating an association with the phospholipids, we expressed and purified the N-terminal segment of JP2 (referred to as JP2^1–452^), see Figure 2(C). JP2^1–452^ binds to PIP strips™ with a similar profile to the full-length protein as shown in Figure 2(D). The intensities of the hybridized spots are noticeably less than those shown in Figure 2(A); this may be partially attributed to the use of a different antibody in order to recognize the N-terminal region of JP2 (ab116077). Using this antibody with the full-length protein also produced weaker intensity bands compared with the antibody for the C-terminus (ab79071) (results not shown). The N-terminal domain interacts most strongly with PtdIns(3,4,5)*P*_3_ and PtdIns(3,5)*P*_2_, but binding to PtdIns(4,5)*P*_2_ is very weak. The truncate also binds to PS, but not with PA, which indicates that the hydrophobic C-terminal domain of the full-length JP2 is involved in mediating this association.

### Characterization of JP2 binding to PS

**Figure 3:**
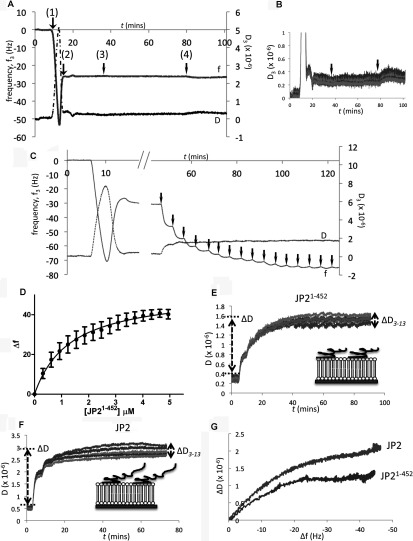
Characterization of JP2 and JP2^1–452^ binding to SLBs containing DOPS (**A**) An exemplar frequency shift time-course plot (*f*/*t*) showing that JP2 does not bind to bilayers containing DOPE and DOPC. (1) Injection of SUVs (DOPC/DOPE/POPC at a ratio of 55:15:30, by vol.) producing a Δ*f* of ~50Hz. (2) Fusion and rupture of the vesicles to form a SLB with a reduction in *f* as water is released and the bilayer is formed, stabilizing at ~30 Hz. (3 and 4) Application, indicated by the black arrows, of two different concentrations of JP2 (200 and 670 nM respectively) does not change the frequency. (**B**) Corresponding *D*/*t* plot shows virtually no change to the overtone spread (*D*_3_–*D*_13_) or magnitude, indicting no JP2 binding. (**C**) Sequential 100 μl (300 nM) additions of JP2^1–452^ to SLBs composed of DOPC/DOPE/POPC/DOPS (55:15:10:20, by vol.), arrows indicate the individual applications of protein. (**D**) Corresponding adsorption isotherm of JP2^1–452^ binding to the SLB. (**E** and **F**) *D*/*t* plots for JP2^1–452^ and full-length JP2 respectively; Δ*D* and spread of the overtones are different for the two proteins. The cartoons (insets) provide a representation of the difference between the size and conformation of JP2 and JP2^1–452^. (**G**) A comparison of the *D*/*f* plots for JP2 and JP2^1–452^ bound to DOPS.

To investigate the interactions of JP2 with lipids in a conformation more akin to the *in vivo* form, we employed the technique of QCM-D using SLBs. Following the method described in [20], SLBs were formed from vesicles composed of either DOPC/DOPE/POPC or DOPC/DOPE/POPC/DOPS at molar ratios of 55:15:30 and 55:15:10:20 respectively. The frequency–time plot in Figure 3(A) shows that JP2 does not bind to supported lipid bilayers composed of phosphatidylethanolamine and PC as there is no change to the frequency, which means no mass has associated with the bilayer. Similarly, the dissipation overtones (Figure 3B) remain virtually unchanged. However, the inclusion of DOPS resulted in binding of both full-length and truncated JP2, with an exemplar plot shown in Figure 3(C). The stepwise addition of JP2^1–452^ leads to a decrease in frequency with each aliquot of protein added, indicating that JP2 is associating with the lipid bilayer. Following the method described in [21] a plot of the change in frequency as a function of the protein concentration generated an adsorption isotherm for both JP2 and JP2^1–452^ binding to DOPS with the profile for the truncate shown in Figure 3(D). From these types of plots the dissociation constant (*K*_d_) could be calculated using a one-site binding model. The N-terminal domain binds to DOPS with a 2-fold lower affinity compared with the full-length protein with a *K*_d_ value of 1.0 μM compared with 0.5 μM (Tables 1 and 2). This quantitative data revealed that the reduction in intensity of the PS spot on the PIP strip™ shown in Figure 2(D) additionally reflects a difference in affinity. The Hill coefficient was calculated to be ~1 for both proteins, which supports the assumption that there is only one DOPS-binding site within each protein molecule and that there is no co-operativity. The addition of JP2^1–452^ results in a smaller change to frequency (although not significantly) consistent with it being a smaller molecule.

**Table 1:** Binding parameters for the interaction of full-length JP2 with SLBs containing DOPS, PtdIns(4,5)*P*_2_ and PtdIns(3,4,5)*P*_3_ Data were analysed using a two-way ANOVA; *P<0.05, **P<0.01, ***P<0.001 compared with JP2 association with DOPC/DOPE/POPC/DOPS (55:15:20:10 molar ratio) in column 1.

Protein	DOPC/DOPE/POPC/DOPS (55:15:10:20, by vol.) JP2	+1% PtdIns(4,5)*P*_2_ (55:15:9:20:1, by vol.) JP2	+1% PtdIns(3,4,5)*P*_3_ (55:15:9:20:1, by vol.) JP2
Δ*f*_max_ (Hz)	46.8±1.7	52.8±3.4	49.4±1.0
Δ*D*	2.5±0.28	3.82±0.10**	4.04±0.13***
Δ*D*_(3–13)_	0.69±0.15	0.72±0.45	1.44±0.04*
*K*_d_ (μM)	0.483±0.054	0.389±0.060	0.335±0.026**
*B*_max_ (Hz)	63.4±2.6	62.6±2.4	58.8±1.1
Hill coefficient	1.1±0.2	1.3±0.2	1.5±0.1

**Table 2:** Analysis of the interaction of the truncated JP2^1–452^ and S101R mutant with SLBs containing DOPS and DOPS plus PtdIns(3,4,5)*P*_3_ Data were analysed using two-way ANOVA; ***P<0.001 **P<0.01 *P<0.05 (^a^values compared with full-length JP2 Table 1, column 1; ^b^values compared with Table 2, column 1; and ^c^values compared with Table 2, column 2).

	DOPC/DOPE/POPC/DOPS (55:15:10:20, by vol.)		+1% PtdIns(3,4,5)*P*_3_ (55:15:9:20:1, by vol.)	
Protein	JP2^1–452^	S101R	JP2^1–452^	S101R
Δ*f*_max_ (Hz)	40.8±3.4	37.3±3.7^a^*	53.2±6.0^b^*	50.3±1.4^c^**
Δ*D*	1.42±0.08^a^**	3.60±0.34^a^***	2.55±0.55^b^*	3.98±0.17
Δ*D*_(3–13)_	0.29±0.06	0.80±0.04	1.21±0.21^b^**	1.21±0.44
*K*_d_ (μM)	0.992±0.08^a^***	0.371±0.031^a^*	0.688±0.024^b^***	0.386±0.017
*B*_max_ (Hz)	51.6±3.5	46.6±0.9	64.3±2.0^b^*	61.5±0.7^c^**
Hill constant	1.0±0.1	1.2±0.3	0.9±0.1	1.2±0.1

The dissipation plots, energy decay profiles over time (*D*/*t*), for the association of JP2^1–452^ and JP2 with DOPC/DOPE/POPC/PS (Figures 3E and 3F) show that the overall change in dissipation (Δ*D*) is greater for the full-length protein compared with the truncate (Tables 1 and 2). The dissipation overtones (*D*_3_–*D*_13_) are also closer together for JP2^1–452^ compared with the full-length protein, which indicates that the truncate forms a more compact structure on the sensor surface as illustrated by the cartoons (Figures 3E and 3F, insets), in keeping with the full-length protein having an additional 244 amino acids. The finding that JP2 adopts a flexible conformation is also consistent with the EM (electron microscopy) images in Figure 1(C) showing how JP2 forms an extended string-like structure. Analysis of Δ*D* against Δ*f* [26] (*D*/*f* plot) provides additional information describing the viscoelastic behaviour of a protein at a given time point providing what may be considered a ‘conformation fingerprint’ characteristic of each adsorbate as a whole. We found that the *D*/*f* plots for each protein binding to a particular SLB were highly reproducible (results not shown). The *D*/*f* plots for both full-length JP2 and JP2^1–452^ in Figure 3(G) reveal that the conformation of the two proteins is different upon association with the bilayer.

### Incorporation of PtdIns(3,4,5)*P*_3_ as well as DOPS into SLBs increases the affinity of JP2 binding

**Figure 4:**
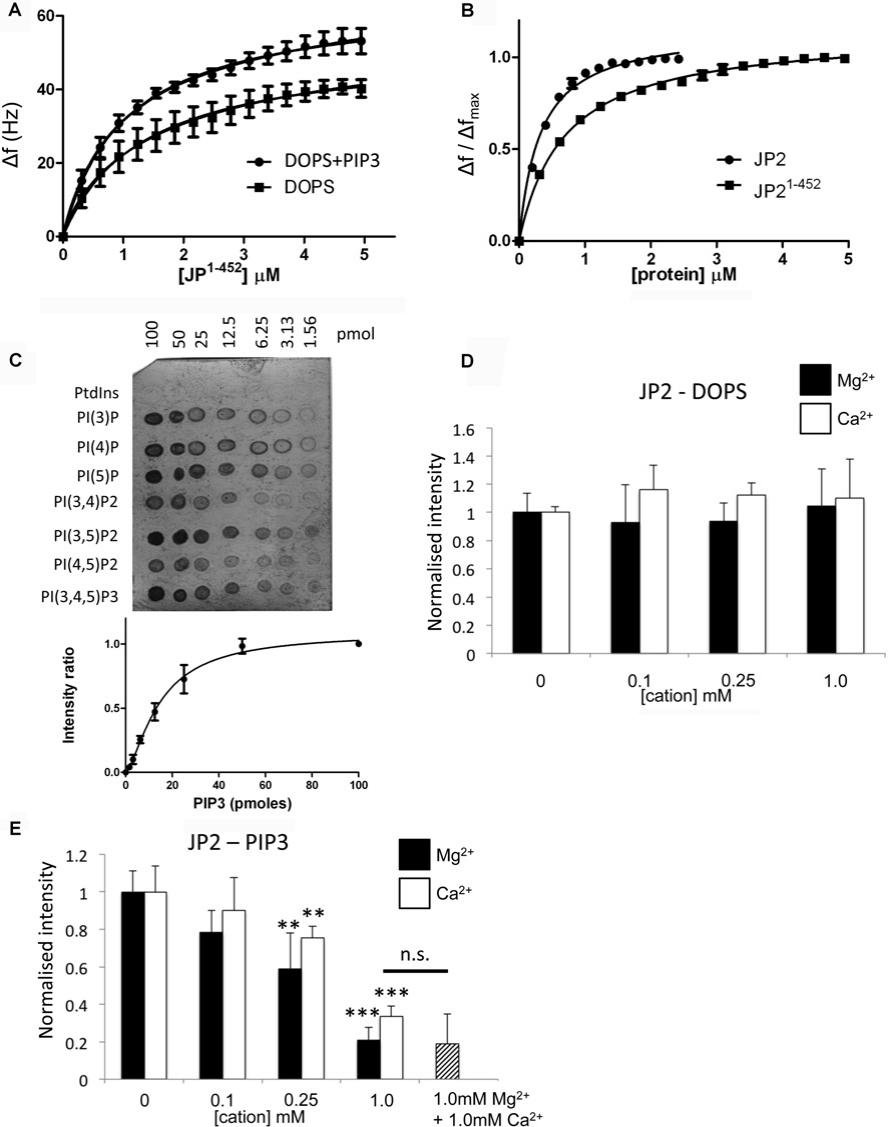
SLBs containing PtdIns(3,4,5)*P*_3_ lead to bound JP2 adopting a more extended conformation (**A**) Adsorption isotherms of JP2^1–452^ binding to SLBs containing DOPS (DOPC/DOPE/POPC/DOPS at a ratio of 55:15:10:20, by vol.) and with PtdIns(3,4,5)*P*_3_ (PIP3) [DOPC/DOPE/POPC/DOPS/PtdIns(3,4,5)*P*_3_ at a ratio of 55:15:9:20:1, by vol.]. (**B**) Adsorption isotherms of full-length and truncated JP2 binding to SLBs containing PtdIns(3,4,5)*P*_3_. (**C**) Example PIP strip™ array showing quantitative binding of JP2 to phosphatidylinositols. A plot of mean spot intensities (*I*/*I*_0_) against PtdIns(3,4,5)*P*_3_ concentration is shown below (*n*=3). PI, PtdIns. (**D**) Densitometric quantification of lipid–protein blots of JP2 binding to DOPS in the presence of increasing concentrations of Ca^2+^ (open bars) and Mg^2+^ (closed bars). (**E**) Analysis of JP2–PtdIns(3,4,5)*P*_3_ blots find a significant reduction in binding when either cation is present in concentrations ≥0.25mM. (**P<0.05, ***P<0.01) *n*=6. n.s., not significant.

The inclusion of PtdIns(3,4,5)*P*_3_ at concentrations mimicking those *in vivo* [27] (DOPC/DOPE/POPC/DOPS/PtdIns(3,4,5)*P*_3_ 55:15:9:20:1, by vol.) resulted in more protein (JP2^1–452^) being absorbed on to the chip, reflected by an increased Δ*f* (*P*<0.05) with the binding isotherms shown in Figure 4(A). Furthermore, as summarized in Table 2, the *K*_d_ value was increased from 0.99 μM to 0.69 μM. Although there is higher affinity binding of the full-length protein when PtdIns(3,4,5)*P*_3_ is present, there is no change to Δ*f*. The binding isotherms for JP2^1–452^ and JP2 presented in Figure 4(B) show that the full-length protein binds with a higher affinity than the truncate. Interestingly, the Hill coefficient for the full-length protein was approximately 1.5; however, the data could not be fitted to a two-site binding model. Since PtdIns(3,4,5)*P*_3_ represents only a fraction of the DOPS-binding sites (1:20 molar ratio) then this was not surprising. Therefore, although we are able to detect an overall increase to the *K*_d_ value, we suggest the reason that the curve is not bimodal is that the high-affinity PtdIns(3,4,5)*P*_3_ binding (illustrated in Figure 4C) is masked by the protein–DOPS binding characteristics and the data cannot be deconvoluted.

### [Ca^2+^] and [Mg^2+^] impair PtdIns(3,4,5)*P*_3_ binding to JP2

Protein–lipid overlay assays revealed that the inclusion of divalent cations in buffers had no effect upon JP2–DOPS interactions (Figure 4D). However, when the [Ca^2+^] or [Mg^2+^] was 0.25 mM or greater there is reduced JP2–PtdIns(3,4,5)*P*_3_ binding, as shown in Figure 4(E). A combination of 1 mM Ca^2+^ in addition to 1 mM Mg^2+^ did not result in any further changes to JP2–PtdIns(3,4,5)*P*_3_ binding.

### PtdIns(3,4,5)*P*_3_ binding to JP2 induces a more flexible conformation of the protein

**Figure 5:**
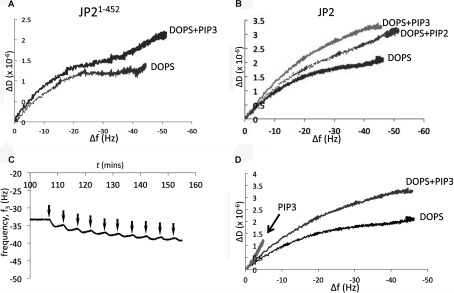
PtdIns(3,4,5)*P*_3_ binding induces a conformational change of JP2 (**A**) *D*/*f* plots of JP2^1–452^ binding to DOPS containing SLBs with and without 1% PtdIns(3,4,5)*P*_3_ (PIP3). (**B**) *D*/*f* plots for JP2 in the absence and presence of PtdIns(3,4,5)*P*_3_ or PtdIns(4,5)*P*_2_ (PIP2) compared with DOPS. (**C**) *f*/*t* plot of JP2 binding to DOPC/DOPE/POPC/PtdIns(3,4,5)*P*_3_ (55:15:29:1, by vol.), arrows indicate addition of JP2. (**D**) *D*/*f* plot illustrating that the conformation of JP2 bound to PtdIns(3,4,5)*P*_3_ is different than JP2 binding to either DOPS or DOPS plus PtdIns(3,4,5)*P*_3_.

The inclusion of PtdIns(3,4,5)*P*_3_ within the SLB resulted in a significant increase to Δ*D* for both JP2 and JP2^1–452^ with a greater spread of the overtones (Δ*D*_3_–Δ*D*_13_) (Tables 1 and 2) with the *D*/*f* plots in Figures 5(A) and 5(B) illustrating there is a conformational change to the protein upon binding to the PtdIns(3,4,5)*P*_3_-containing bilayer. Both JP2 and JP2^1–452^ adopt a more extended and flexible conformation in the presence of PtdIns(3,4,5)*P*_3_. Analysis of the dissipation properties of bilayers containing DOPS and both DOPS and PtdIns(3,4,5)*P*_3_ (20:1, v/v) in the absence of protein found that there was no significant difference to the viscoelastic properties; Δ*D*_3_–Δ*D*_13_ was measured, *n*=6, as 0.27±0.16 and 0.40±0.10 respectively so we can rule out the bilayer composition differences as contributing towards the change to the protein dissipation profiles. Although the PIP strips™ (Figure 2) show that JP2 exhibits weak binding to PtdIns(4,5)*P*_2_, we also examined JP2 binding to bilayers containing PtdIns(4,5)*P*_2_, since this phospholipid is most abundant in the mammalian cell membrane [27]. The results in Table 1 reveal that there is a significant increase to Δ*D*, but the spread of the overtones (Δ*D*_3_–Δ*D*_13_) remains unchanged compared with bilayers with DOPS only (i.e. DOPC/DOPE/POPC/DOPS at a ratio of 55:15:10:20, by vol.). The inclusion of 1% PtdIns(4,5)*P*_2_ did not change the *K*_d_ value (see Table 1). However, the *D*/*f* plots in Figure 5(B) do show that JP2 undergoes a conformational change upon inclusion of PtdIns(4,5)*P*_2_, but the increase in viscoelastic properties are greater for PtdIns(3,4,5)*P*_3_.

The Δ*D* value for each bilayer composition will be representative of all the bound JP2 molecules and so when there is both DOPS and PtdIns(3,4,5)*P*_3_ [or PtdIns(4,5)*P*_2_] present there will be different populations of JP2, which cannot be deconvoluted. Bilayers containing only 1% PtdIns(3,4,5)*P*_3_ but no DOPS (DOPC/DOPE/POPC/PtdIns(3,4,5)*P*_3_ at a ratio of 55:15:29:1, by vol.) revealed that JP2 binding led to a Δ*f* of 5.7±0.4 Hz (see *f*/*t* plot in Figure 5C). This relatively small Δ*f* is in keeping with there only being a few potential binding sites for JP2 compared with a bilayer composed of 20% DOPS and so we cannot directly compare the absolute values for Δ*D* and Δ*D*_3_–Δ*D*_13_. The *D*/*f* plots shown in Figure 5(D) do, however, show that the slope of JP2 binding to 1% PtdIns(3,4,5)*P*_3_ is much steeper compared with SLBs containing both PtdIns(3,4,5)*P*_3_ and DOPS, suggesting that the bound protein has adopted a different conformation. SUVs containing a higher concentration of PtdIns(3,4,5)*P*_3_ did not form stable bilayers, making a direct comparison with DOPS-binding parameters not possible using QCM-D.

### JP2 binds PtdIns(3,4,5)*P*_3_ and PS at two different sites

**Figure 6:**
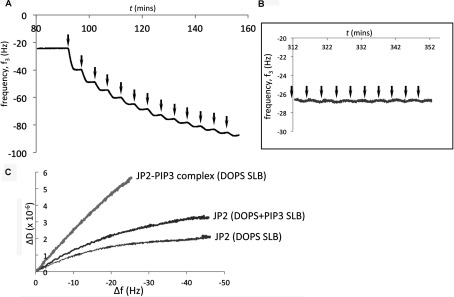
JP2 contains a separate PtdIns(3,4,5)*P*_3_- and PS-binding site (**A**) *f*/*t* plot of a complex of JP2–PtdIns(3,4,5)*P*_3_ (1:1 molar ratio; [JP2]=200 nM) applied to SLBs (DOPC/DOPE/DOPS, 65:15:20, by vol.). (**B**) Control experiments with soluble PtdIns(3,4,5)*P*_3_ (100 μl aliquots, 200 nM solution) shows it does not bind to SLBs. (**C**) *D*/*f* plot illustrating that the JP2–PtdIns(3,4,5)*P*_3_ (PIP3) complex bound to DOPS adopts a highly flexible conformation.

The results described above suggest that JP2 adopts a different conformation when bound to PtdIns(3,4,5)*P*_3_ or DOPS, thus we next explored whether JP2 binding to each phospholipid was mutually exclusive. Purified JP2 was incubated with soluble PtdIns(3,4,5)*P*_3_ (1:1 molar ratio), to form a JP2–PtdIns(3,4,5)*P*_3_ complex which was then applied to SLBs composed of DOPC/DOPE/DOPS (65:15:20, by vol.). If PtdIns(3,4,5)*P*_3_ occupies the same binding site as DOPS then we would not expect to see an interaction with the SLBs. However, the *f*/*t* plot in Figure 6(A) shows that the JP2–PtdIns(3,4,5)*P*_3_ complex bound to the DOPS bilayers. Control experiments showed that soluble PtdIns(3,4,5)*P*_3_ alone did not bind to the bilayer as shown in Figure 6(B). The *D*/*f* plot in Figure 6(C) further reveals that the JP2–PtdIns(3,4,5)*P*_3_ complex bound to DOPS adopts a more flexible conformation with a larger Δ*D*.

### The HCM S101R mutation results in a conformational change to the protein structure

**Figure 7:**
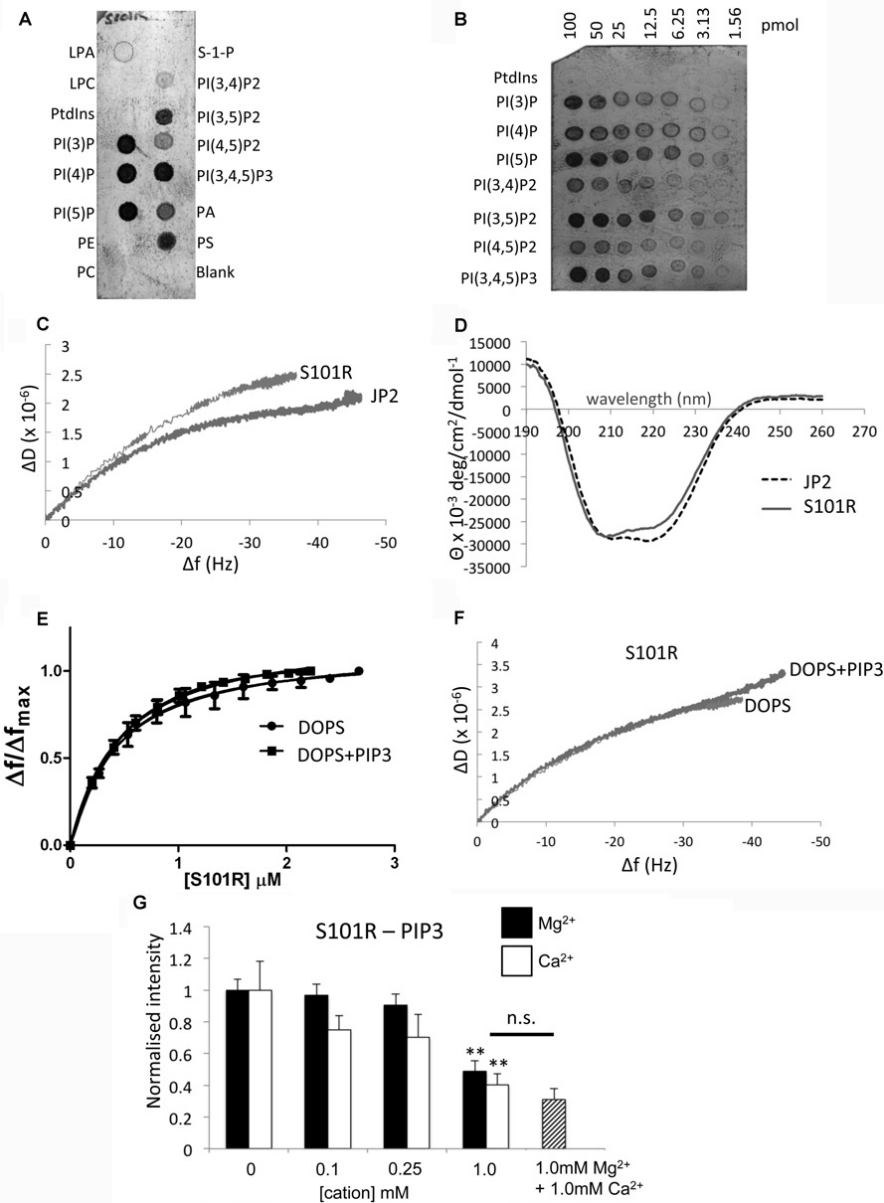
Characterization of S101R–phospholipid interactions (**A** and **B**) PIP strip™ and PIP array of S101R phospholipid binding. (**C**) *D*/*f* plot for S101R and JP2 binding to DOPC/DOPE/POPC/DOPS illustrating the two proteins adopt a different conformation. (**D**) CD spectra of S101R and wild-type JP2 illustrating that the amino acid change has resulted in modification of the secondary structure. (**E**) Adsorption isotherm of S101R binding to SLBs without PtdIns(3,4,5)*P*_3_ (DOPC/DOPE/POPC/DOPS at a ratio of 55:15:10:20, by vol.) and with PtdIns(3,4,5)*P*_3_ [DOPC/DOPE/POPC/DOPS/PtdIns(3,4,5)*P*_3_ at a ratio of 55:15:9:20:1, by vol.). (**F**) *D*/*f* plots for S101R binding to SLBs in the presence and absence of PtdIns(3,4,5)*P*_3_. (**G**) Densitometric quantification of lipid–protein blots of JP2–PtdIns(3,4,5)*P*_3_ interactions in the presence of increasing concentrations of Ca^2+^ (open bars) and Mg^2+^ (closed bars), *n*=6 (**P<0.05). LPA, lysophosphatidic acid; LPC, lysophosphocholine; n.s., not significant; PE, phosphatidylethanolamine; PI, PtdIns; S-1-P, sphingosine 1-phosphate.

In order to investigate whether the single amino acid substitution, near MORN domain 4, impacts upon phospholipid binding we engineered, using site-directed mutagenesis, the S101R mutation and expressed and purified the mutant protein in *E. coli* cells. Yields and purity were very similar to those for the wild-type JP2 as shown by SDS/PAGE in Figure 1(B). Examination of the purified protein by TEM did not find, at these resolutions, any difference in particle size and shape compared with the wild-type samples. We also found that the interaction of S101R mutant with phospholipids produced binding profiles virtually identical to the wild-type JP2 as shown in Figures 7(A) and 7(B) (compare with Figures 2A and 4C). However, QCM-D studies revealed that the introduction of the mutation led to an increased binding affinity to PS, as reported in Table 2. Other differences between the way in which S101R and wild-type JP2 interacted with the DOPS SLB were that less protein was adsorbed on to the chip with a corresponding lower *B*_max_ (*P*<0.01).

Intriguingly, the *D*/*f* plot (Figure 7C) revealed that the conformation of the mutant protein upon binding to DOPS is modified compared with wild-type JP2 and is reflected by an increased Δ*D* and larger spread of the overtones as reported in Table 2. These data indicate that the mutation has resulted in a structural reorganization of the protein and that it has formed a more flexible layer upon binding to the SLB (DOPC/DOPE/POPC/DOPS). In an alternative approach to probe the structure of S101R compared with the wild-type protein, we also employed CD to provide secondary structure information, with the resulting spectra shown in Figure 7(D). A change to the helical and β-sheet content was measured as reported in Table 3.

**Table 3:** CD spectra analysed for secondary structure content using DICROWEB Secondary structure is given as a percentage of the total protein. NRMSD, Normalized RMSD.

Protein	Helix	Sheet	Turns	Unordered	NRMSD
JP2	38	16	18	28	0.014
S101R	24	20	22	28	0.026

Unlike wild-type JP2 or JP2^1–452^, the introduction of PtdIns(3,4,5)*P*_3_ to the SLB composition did not result in a change to the binding constant for S101R (Table 2), as shown in Figure 7(E), but yet more protein bound to the bilayer (larger Δ*f*). Significantly, it can be seen that an interaction of S101R with PtdIns(3,4,5)*P*_3_ did not elicit a change to the protein conformation fingerprint (see the *D*/*f* plot in Figure 7F). The lipid blots in Figures 7(A) and 7(B) indicate that S101R binds PtdIns(3,4,5)*P*_3_, and since using QCM-D we find an increase in frequency when PtdIns(3,4,5)*P*_3_ is present in the SLB we interpret the data to mean that PtdIns(3,4,5)*P*_3_ is able to bind to S101R, but this interaction does not confer a structural rearrangement to the mutant protein, possibly as it is already in an extended form. We additionally determined that, as reported for the wild-type protein, Ca^2+^ and Mg^2+^ had no effect upon the S101R–DOPS interactions (results not shown). Similarly, the presence of either of the cations reduced S101R binding to PtdIns(3,4,5)*P*_3_. However, concentrations of 1 mM were required to achieve a significant reduction in S101R–PtdIns(3,4,5)*P*_3_ association, as shown in Figure 7(G).

## DISCUSSION

In the present study we provide the first robust characterization of the lipid-binding properties of JP2. Taken together with the TEM data we are now able to show how monomeric JP2 forms a rod-like structure with dimensions that are compatible with a protein linking two membranes separated by 12–15 nm. In this form the JP2 filaments with a diameter of roughly ≤2 nm would not be readily resolved in tomograms of the dyadic cleft (e.g. [28]) and so explains why JP2 densities have not been previously described. Additionally, we have advanced our understanding of how the introduction of a single point mutation identified in patients with HCM may affect the function of the protein by incurring changes to the structural and lipid-binding properties.

Our data show that the N-terminal region of JP2 mediates membrane adhesion, although the full-length protein binds with an approximately 2-fold higher affinity. This may be due to regions of the full-length protein that help stabilize the N-terminal domain in a conformation that promotes lipid binding. We also cannot rule out that some full-length JP2 molecules may bind to the bilayer via the C-terminal region, which would influence the binding profile, although if this were the case then we would have expected to find a greater variability between the datasets for the full-length protein, which was not the case. A significant finding from the present study is that JP2 binds specifically to PS, in keeping with PS being normally localized to the inner leaflet of the sarcolemma. Since there is only weak binding of JP2 to DOPG (1,2-dioleoyl-*sn*-glycero-3-phosphoglycerol) this implies that the molecular basis for JP2 binding to PS is not purely an electrostatic interaction between the negatively charged lipid headgroups with the cationic protein residues, since DOPG has the same overall net charge of −1 at physiological pH. Interestingly, under conditions of cell apoptosis, PS is found to translocate from the inner to outer leaflet of the plasma membrane [29]. Externalization of PS is widely recognised as a hallmark of stressed or dying cells and has been associated with cardiovascular diseases as well as during ischaemia–reperfusion [30]. Given the strong specificity of JP2 for PS, disruption of PS asymmetry may be a hitherto unidentified factor that contributes towards the loss of dyad structures observed in heart failure, and, in the aged heart, resulting in impaired JP2 binding to the t-t and independent of changes to protein expression levels. Low sub-micromolar binding constants for JP2–PS interactions are in the range reported for other proteins with membrane adhesion properties, e.g. PTEN (phosphatase and tensin homologue deleted on chromosome 10) protein [a phosphatase that hydrolyses PtdIns(3,4,5)*P*_3_ to PtdIns(4,5)*P*_2_] has calculated equilibrium binding constants of ~12 μM and 0.4 μM for PS and PtdIns(4,5)*P*_2_ respectively [31].

The PIP strip™ analysis showed that full-length JP2 binds most strongly to the monophosphate inositols. Although the monophosphates are generally considered to be intermediate phosphoinositide metabolites, there is evidence to suggest that they function as signalling molecules regulating processes such as vesicular trafficking [32]. However, the different physiochemical properties of the phosphoinositols result in differences to solubility and stability upon adsorption on to nitrocellulose so that the monophosphates often give the strongest signal on protein–lipid overlay assays. The water solubility of the diphosphates are all similar, but are greater than the monophosphates [33]. Therefore the differences in intensity on the PIP strip™ can be taken to indicate a selectivity for PtdIns(3,5)*P*_2_ and PtdIns(3,4,5)*P*_3_ compared with PtdIns(4,5)*P*_2_ and PtdIns(3,4)*P*_2_, which would suggest that the MORN domains adopt a structural fold, a binding pocket. These data also indicate that the 3,5-phosphate groups influence the association. Proteins that exhibit specificity for phophatidylinositols are often characterized by the presence of one or more PH (pleckstrin homology) domains or FYVE, PX (Phox homology), ENTH (epsin N-terminal homology), CALM (clathrin assembly lymphoid myeloid), PDZ, PTB (phosphotyrosine-binding) and FERM (4.1/ezrin/radixin/moesin) motifs [34,35]. Analysis of the JP2 primary sequence did not identify any of these motifs; however, there is often minimal sequence homology between individual PH motifs, although the 3D structures are conserved, making the presence of phosphoinositide-binding domains difficult to predict. Therefore we cannot rule out the possibility that several of the MORN domains (predicted to form β-sheets) adopt, for example, a pseudo-PH-like structure.

Interestingly, it has been reported that the t-t morphology is disrupted in a PI3K (phosphoinositide 3-kinase)-knockout mouse model [36] and, although the expression of JP2 at the protein level was not altered, a population of JP2 was identified that was not co-localized with RyR2, which led the authors to conclude that PtdIns(3,4,5)*P*_3_ is required for the correct targeting of JP2. The results from the present study show that the inclusion of PtdIns(3,4,5)*P*_3_ at levels mimicking those expected to be found *in vivo* does marginally increase the overall equilibrium binding constant for JP2, although we also show that JP2 binds to PtdIns(4,5)*P*_2_. Our data also indicate that phosphoinositides are not required for adhesion of JP2 to the membrane, as the more abundant PS orchestrates binding. However, these data would not preclude a role for PtdIns(3,4,5)*P*_3_ in the targeting of JP2 to the dyadic cleft. In the mammalian cell, PtdIns(4,5)*P*_2_ is normally more abundant than PtdIns(3,4,5)*P*_3_ with PtdIns(3,5)*P*_2_ at levels approximately 100-fold lower compared with PtdIns(4,5)*P*_2_ [26], and so a role for a JP2–PtdIns(4,5)*P*_2_ interaction cannot be ruled out, although the action of PI3K will lead to temporal and spatial increases in local concentrations of PtdIns(3,4,5)*P*_3_. Our experiments show that there is a binding site for phosphatidylinositols that is separate from that for PS, which means that the JP2–phosphatidylinositol interaction has the potential to be relevant *in vivo* during stages of phosphoinositide metabolism.

Our other major finding from the present study is that the presence of PtdIns(3,4,5)*P*_3_ and to a lesser extent PtdIns(4,5)*P*_2_ results in a different JP2 conformation compared with the form bound to DOPS. PtdIns(3,4,5)*P*_3_-induced conformational transitions have been reported for other proteins with binding via an association with PH domains (e.g. [37]). The EM data in the present study provides the first structural information for JP2 from which it can be seen how the molecule forms a filament and thus could conceivably undergo conformational changes. Taken together, these data provide a novel insight as to how JP2 may act as a linker molecule and also respond to dynamic changes to the cell dimensions that occur during excitation–contraction coupling which conceivably requires some level of protein flexibility to combat movement of the fluidic membranes and mechanical forces generated by the contraction cycle. A PtdIns(3,4,5)*P*_3_-induced structural rearrangement may also be instrumental for exposing, or occluding, potential JP2–protein binding sites that mediate transient interactions with other molecular components within the dyadic cleft and thus allow JP2 to act as an indirect regulator of Ca^2+^ cycling.

Modelling studies predict that at the end of Ca^2+^ release from the jSR, there are millimolar Ca^2+^ concentrations at the centre of the cleft [38]; although generally it is assumed to be in the range of 100 μM. Additionally, phospholipids at the sarcolemma are also proposed to bind a large portion of the free Ca^2+^ at the termination of jSR release. Therefore it is not clear whether rapid local temporal increases in [Ca^2+^] as a result of CICR within the dyad compartment will affect JP2–PtdIns(3,4,5)*P*_3_ interactions, as we have shown that [Ca^2+^] must approach 0.25 mM for binding to be impaired. However, this Ca^2+^ dependency may become more significant in pathological conditions that lead to Ca^2+^ overload in some cardiovascular disorders [39]. Protein—PtdIns(4,5)*P*_2_ interactions modulated by Ca^2+^ concentrations have been described previously [27]. During diastole, levels of Mg^2+^ are 0.5–1.2 mM [40,41] and so the modulation of JP2–PtdIns(3,4,5)*P*_3_ binding by [Mg^2+^] shown in the present study is potentially physiologically relevant. The fact that divalent cations (Mg^2+^ and Ca^2+^) modulate JP2–PtdIns(3,4,5)*P*_3_ binding but not JP2–DOPS binding also lends further support to there being distinct structural determinants for phospholipid binding. The data also suggests that there is only one divalent cation-binding site.

The introduction of the S101R mutation increased the affinity of the protein for DOPS, which implies that the residue forms part of, or is close to, the lipid-binding domain; the introduction of a basic positively charged arginine residue in place of the neutral serine amino acid may promote an electrostatic interaction with the bilayer. However, less protein bound to the SLBs, which we suggest may be due the modified conformation resulting in steric hinderance and occlusion of some of the DOPS-binding sites. The finding that the conformation of S101R is not modulated by PtdIns(3,4,5)*P*_3_ may have implications for stabilization of the jSR/t-t configuration. The S101R mutation is believed to be a causative agent in the pathology of HCM, although an engineered N101R mouse equivalent when expressed in HC92 cells did not result in significant hypertrophy, but was characterized by depressed sarcoplasmic reticulum Ca^2+^ release [14]. Therefore, we suggest that the structural changes identified in the present study may be responsible for altered JP2–protein interactions. Currently, little is known about JP2's binding partners, although data is emerging to suggest a JP2–triadin association [42] and a JP1–RyR1 interaction has been described in skeletal muscle [43]. In conclusion, in the present study we have systematically analysed the lipid-binding properties of wild-type JP2 and the S101R mutant with the findings offering new insights into the role of this protein in dyad organization and how changes to the structural characteristics of the JP2 may play a role in the pathogenesis of disease.
